# Crack Propagation and Fatigue Performance of Partial Posterior Indirect Restorations: An Extended Finite Element Method Study

**DOI:** 10.3390/jfb14090484

**Published:** 2023-09-21

**Authors:** Mehmet Gökberkkaan Demirel, Reza Mohammadi, Murat Keçeci

**Affiliations:** 1 Department of Prosthodontics, Faculty of Dentistry, Necmettin Erbakan University, Konya 42090, Turkey; 2 Faculty of Dentistry, Necmettin Erbakan University, Konya 42090, Turkey; reza.mohammadi@ogr.erbakan.edu.tr; 3 Department of Prosthodontics, Faculty of Dentistry, Karamanoğlu Mehmet Bey University, Karaman 70000, Turkey; muratkececi@kmu.edu.tr

**Keywords:** an extended finite element method, CAD-CAM restorations, dental digital technologies, prosthodontics

## Abstract

Dental ceramics are susceptible to slow, progressive crack growth after cyclic loading. The purpose of this study was to investigate the progressive patterns of cracks in two different types of CAD/CAM ceramic materials used with three different partial posterior indirect restoration (PPIR) designs and to determine the materials’ failure risk using a fatigue test. Standard initial cracks were formed in Standard Tessellation Language (STL) files prepared for three different PPIRs. The materials chosen were monolithic lithium disilicate (LS) and polymer-infiltrated ceramic networks (PICNs). The extended finite element method (XFEM) was applied, and the fatigue performance was examined by applying a 600 N axial load. The cracks propagated the most in onlay restorations, where the highest displacement was observed. In contrast, the most successful results were observed in overlay restorations. Overlay restorations also showed better fatigue performance. LS materials exhibited more successful results than PICN materials. LS materials, which can be used in PPIRs, yield better results compared to PICN materials. While inlay restorations demonstrated relatively successful results, overlay and onlay restorations can be specified as the most and the least successful PPIR types, respectively.

## 1. Introduction

Clinicians currently use direct, semi-direct, semi-indirect, and indirect techniques for the rehabilitation of missing tooth tissues. When using direct restorations, there are challenges, such as restoring the occlusal anatomy (particularly in the posterior region), ensuring marginal adaptation, and ideally preparing contours [1]. In particular, polymerization shrinkage creates major concerns in the long term. When the dentin–resin hybrid layer encounters contraction stresses, it can create gaps; this is because the decrease in bond strength and the use of strong adhesives can cause cuspal deflection and enamel fractures at the level of the cusp [2].

Cuspal deflection is a biomechanical phenomenon characterized by the linear displacement of the cusp tips of a tooth due to the interplay between the polymerization shrinkage stress of the composite material and the flexibility of the cavity wall. This phenomenon has been observed to range from approximately 10 µm to 45 µm during composite restoration, with variations attributed to the measurement technique, tooth morphology, and cavity dimensions [3]. The application of thick layers of luting material may result in shrinkage strain and additional stress during the polymerization process. These stresses have the potential to diminish bond strength, elevate the likelihood of debonding, and ultimately shorten the lifespan of the restoration [4]. Even though layering techniques, fiber patches, and different polymerization procedures have been introduced [2] to decrease stresses while using the direct technique, the most effective method, with which polymerization shrinkage in mesio-occluso-distal (MOD) restorations with especially wide cavities can be best controlled, is to use “luted” partial posterior indirect ceramic restorations (PPIRs), as they create a very limited occurrence of shrinkage in a very thin layer [5,6] and cuspal coverage restorations could help prevent extreme cuspal deflection [7].

Another major advantage of the restorations produced with the indirect technique is that they are highly conservative options that ensure the protection of intact tooth tissue [8]. In full-coverage crown preparation, a significant reduction of enamel and dentin tissue (50–68%) is required, whereas the most substantial reduction in PPIR preparation occurs in overlay restorations (35–38%). This allows for the preservation of a greater amount of healthy tooth structure. [9]. A preparation design necessitating the extensive removal of hard tooth structure should be avoided for several reasons, including the fact that it leads to the exposure of dentin in close proximity to the pulp, with a high density of dentinal tubules resulting in an elevated secretion of dentinal fluid and increased risk of postoperative sensitivity. This, in turn, adversely affects the ratio of remaining dentin to cavity size, including the pulp cavity and dentin tubules. Furthermore, it heightens the risk of postoperative sensitivity and is contraindicated for young patients with large pulp chambers When indirect ceramic restorations are applied, pulp is protected through the application of more minimal preparation types (PPIR inlay, onlay, and overlay), and an increase in the longevity of the tooth is expected with increased dental tissue as a result of care exercised in minimally invasive dentistry [10].

Although monolithic lithium disilicate (LS) ceramic materials have been used successfully in PPIR for a long time, polymer-infiltrated ceramic network (PICN) materials are increasingly used for clinical purposes. LS materials have long been an excellent option in PPIR applications, with their optimal aesthetics and adequate resistance properties [11]. However, it has been reported that in regions with brittle structures and occlusal stresses, their disadvantages—such as dispersion in the tooth tissues and fractures or cracks that could occur in the restoration—might have catastrophic effects on the tooth tissue [12,13,14]. PICN materials were produced through the infiltration of polymerizable monomers into partially sintered porous ceramics, followed by heat-induced polymerization for curing. This results in the formation of two interpenetrating networks, namely the polymer and ceramic phases [15].

In clinical trials assessing all-ceramic restorations, it has been revealed that restoration cracks stand out as a primary cause of failure [16]. Cracks may emerge as a result of plastic deformations in high-stress peaks or stress concentrations in a specific region, as occurs with structural and production defects [17]. During load, stress that emerges in these regions is not distributed homogenously in the restoration [18]. As a result of the stress concentration occurring in the defective region, cracks progress and permanent damage emerges in the mechanical element [19,20]. Conditions that may emerge as a result of multiple loading variation through three-dimensional finite element analysis (FEM) are set forth by definite calculations, and even the behaviors of very complex geometrical structures can be interpreted [21]. The extended finite element method (XFEM), which is an effective method for understanding the fracture mechanism and estimating when material might be unsuccessful, is also a useful tool for analyzing the progression of a crack occurring due to pre-existing defects [22]. An initial crack can be placed inside the element related to this method, and as a result of the analysis, the progression of the crack and consequent final fracture risk calculation can be performed. As this method forms discontinuous regions along the crack and around its tip, a better dispersion pattern can be ensured by not remeshing and increasing the degree of freedom [17,20]. Therefore, XFEM is a useful method for analyzing the mechanical behavior of restoration material fractures and estimating their failure risk.

Dental ceramics are susceptible to slow, progressive crack growth after cyclic loading in the oral environment [23]. Fatigue resistance is an important factor that determines the longevity of ceramic restorations [24]. Despite the fact that, in previous studies [11], good results have been reported for LS materials regarding fatigue resistance for over a decade, studies on fatigue resistance for PICNs have only been reported in the last few years. In addition, the results of studies comparing the fatigue resistance of the two materials are not consistent [14,25,26,27,28], and conducting more studies will be helpful in yielding more definite results.

To address these issues, the purpose of this XFEM study was to investigate the progressive patterns of cracks occurring in two different computer-aided design/computer-aided manufacturing (CAD/CAM) ceramic materials used in three different PPIR designs and calculate their failure risk using a fatigue test. The initial hypothesis of the study was that the restoration types and materials would not affect the crack progression pattern or failure risk.

## 2. Materials and Methods

### 2.1. Finite Element Modeling

**Figure 1:**
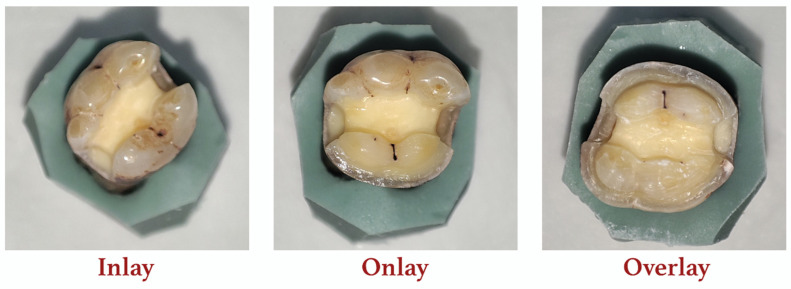
Preparation of different PPIR types.

For this study, an intact no. 36 tooth free of caries was stored in saline solution for a week. Thereafter, it was embedded in a silicone mold and inlay, onlay, and overlay preparations were performed. With regards to the crown, the buccolingual, mesiodistal, and cervico-occlusal dimensions were 11.9 mm, 9.8 mm, and 7.8 mm, respectively. For the inlay, the cavity design had a mesiodistal length of 11.6 mm between the proximal margin edges, with an isthmus width of 3.2 mm. From the mesiobuccal cusp to the cavity floor, the length was 4.6 mm, and proximal boxes with a depth of 3.2 mm were created on both mesial and distal surfaces from the cavity floor to the cemento–enamel junction. The inner buccal and lingual walls were characterized by an 8° taper. With regards to the onlay form, the inlay form modified with a 2.5 mm occlusal reduction on the lingual cusps and a chamfer margin was created 3.6 mm above the cemento–enamel junction on the lingual surface. The axial walls on the lingual surface were characterized by a 6° taper. For the overlay form, the onlay form modified with a 2.5 mm occlusal reduction on the buccal cusps and a chamfer margin was created 3.8 mm above the cemento–enamel junction on the buccal surface. The axial walls on the buccal surface were characterized by a 6° taper (Figure 1). The data were transferred to a design program after being scanned with a model scanner (Vinyl High Resolution, Smart Optics, Bochum, Germany) following each phase. Margin points were determined in the design program (Dental Cad 3.1 Rijeka, EXOCAD, Darmstadt, Germany), and inlay, onlay, and overlay restorations with the suitable anatomical forms were designed with the same data. The design files obtained were extracted in Standard Tessellation Language (STL) format, and required adjustments were performed after they were transferred to a suitable program (Geomagic Design X 2020/0.3) (3D systems, Morrisville, NC, USA) to eliminate errors and make them compatible with finite element analysis.

The 3D image was segmented into distinct surfaces using the Geomagic Design X 2020.0 software (3D systems, Morrisville, NC, USA) to generate appropriate configurations for XFEM. For simplicity in handling the intricacies of three-dimensional finite element models, the enamel, dentin, and dental materials were considered to possess isotropic and linearly elastic properties. Given that the periodontal ligament (PDL) was not incorporated into the model, fixed and pinned boundary conditions were implemented to simulate the constraint of tooth roots within the bone. Specifically, a solitary tooth and corresponding tooth type were employed in the analysis, excluding any representation of the periodontal ligament or bone structure.

The Standard for the Exchange of Product Model Data (STP) files, which were meticulously prepared, underwent seamless transfer to finite element analysis software, specifically ABAQUS (2020 Dassault Systems Simulation Corp., Johnston, RI, USA). The initiation of the crack was meticulously orchestrated, featuring a designated length of 10 µm and a correspondingly slender thickness of 0.01 µm. This precise configuration was meticulously integrated into the ABAQUS software’s component section, whereby the crack was positioned with its shorter edge strategically oriented towards the central pit within the occlusal fossa of the restoration. The mechanical properties attributed to LS and PICN materials, gleaned from comprehensive antecedent investigations and tabulated in Table 1, served as the foundation for the restoration’s material specifications. Subsequently, the carefully formulated scenario was implemented, guided by a meticulous and systematic approach that upholds the standards of empirical rigor and scientific precision. The appropriate mechanical boundary conditions were established using the ‘create boundary condition’ tab within the load segment of the ABAQUS software. Acknowledging the absence of the periodontal ligament’s influence, the tooth was constrained by pinning (U1 = U2 = U3 = 0) from the enamel–cementum junction to the apical region. An isotropic linear elastic simulation was conducted for the restorative materials. To exert a 600 N load, the necessary pressure was computed based on the surface area of each model. XFEM was then performed using the ABAQUS software to evaluate stress distribution.

Occlusal loading was designed and applied as 600 N, which is accepted as the average chewing force in the axial direction. The materials’ properties are provided in Table 1. All materials were accepted as isotropic, linear, and elastic. Additionally, the effects of periodontal ligaments were ignored, and roots were considered to be embedded inside the bone.

### 2.2. An Extended Finite Element (XFEM) Analysis

In the present study, XFEM fracture analysis was conducted to simulate the initiation and propagation of cracks in inlay, onlay, and overlay restorations using ABAQUS (ABAQUS, 2020 Dassault Systems Simulation Corp., Johnston, RI, USA). The cracking criterion used was the maximum principal stress as defined by

(1) $$f^{e}=\frac{\sigma_{1}^{e}}{\sigma_{\max}^{0}}$$

where σ^0^_max_ refers to the allowable maximum stress (fracture strength) of the CAD/CAM materials, $\sigma_{1}^{e}$ is the first principal stress in element (e), and $f^{e}$ represents the stress ratio, which determines whether cracking will occur in the element. A crack was assumed to occur when the maximum principal stress in tension exceeded the predefined tensile strength of the material.

The energy-based damage evolution criterion was applied. It can be defined as a function of the mixed-mode crack initiation using a tabulated form of the power law criterion, as summarized in the following equation [29]:

(2) $${\left(\frac{G_{I}}{G_{IC}}\right)}^{\alpha }+{\left(\frac{G_{II}}{G_{IIC}}\right)}^{\alpha }+{\left(\frac{G_{III}}{G_{IIIC}}\right)}^{\alpha }=1$$

where G_I_, G_II_, and G_III_ represent the energy release rates of crack extension modes I, II, and III, respectively. G_IC_, G_IIC_, and G_IIIC_ are the predefined critical energy release rates of modes I, II, and III, respectively. In this study, the power was determined as α = 1 [30].

The strain energy release rate was measured using the Irwin fracture condition, expressed as follows:

(3) $$G_{I}=G_{II}=G_{III}=\frac{K_{IC}^{2}}{E}$$

### 2.3. Calculation of Fatigue Performance

The progression of the damage in the materials used was separated into three phases. In the first phase, small cracks formed in different locations of the matrix, and damage accumulated rapidly. In the next phase, matrix and fiber separation accompanied the slow and gradual increase in damage. Finally, in the third phase, damage increased rapidly, and fiber breakage was the main mechanism directing this phase.

Stiffness-based methods can simulate all these phases. They are applied by measuring the changes in the elastic modulus of the material. The value of the elastic modulus at the instant of fracture is not equal to zero. Consequently, the damage parameter is associated with the changes in the elastic modulus and cycle ratio [31].

(4) $$D(n)=\frac{E_{0}-E\left(n\right)}{E_{0}-E_{f}}=1-{\left(1-{\left(\frac{n}{N}\right)}^{B}\right)}^{A}$$

In this equation, $E_{0}$ is the static Young’s modulus, $E_{f}$ is the fracture Young’s modulus, E (n) is the Young’s modulus of the cycle, n is the cycle count included in the analysis, N is the final fracture cycle, and A and B are the properties of the material used [31].

(5) $$\begin{array}{l} B=C_{1}\frac{\log N}{\left(1-R)(\frac{\sigma_{\max}}{\sigma_{r}}\right)}\\ A=0.85B+0.42 \end{array}$$

In this equation, $\sigma_{\max}$ is the maximum stress, $\sigma_{r}$ is the residual stress, and $R=\frac{\sigma_{\min}}{\sigma_{\max}}$ [32].

(6) $$\sigma_{r}=\sigma_{u}-(\sigma_{u}-\sigma_{\max})(\frac{n}{N})$$

## 3. Results

**Figure 2:**
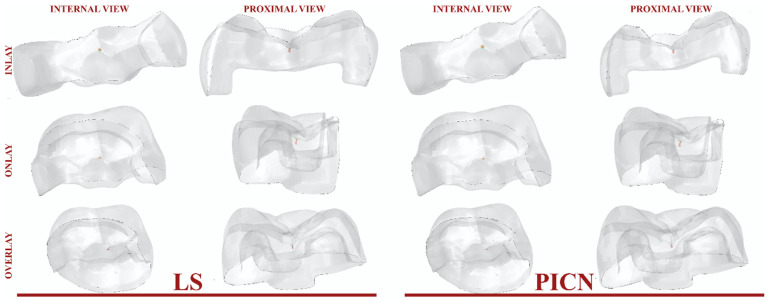
Crack propagation as it occurs in different PPIR types.

The propagation of the cracks for PPIR designs is presented in Figure 2.

**Figure 3:**
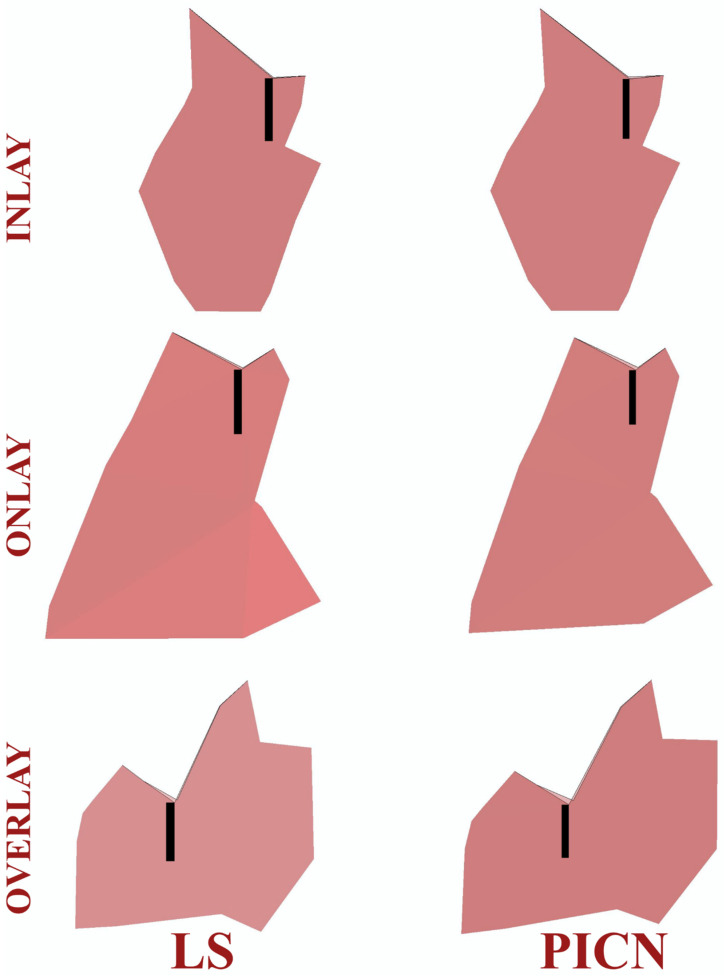
Propagation of cracks and initial crack nucleation from a proximal view.

Crack propagation in all the investigated models was found to be consistent, in which cracks were propagated with increasing cycles. Representative illustrations of the initial cracks (in black) and the final crack pattern (in pink) are depicted in Figure 3. In the PPIR designs, the crack originated on the occlusal surface from the base of the initial crack. It then propagated along the vertical and horizontal direction. According to the results of this study, while crack propagation followed different paths in different PPIR designs, similar patterns were exhibited in different materials with the same designs. Crack propagation patterns predominantly followed a vertical path for inlay designs, a horizontal path for overlay designs, and both horizontal and vertical paths for onlay designs.

**Figure 4:**
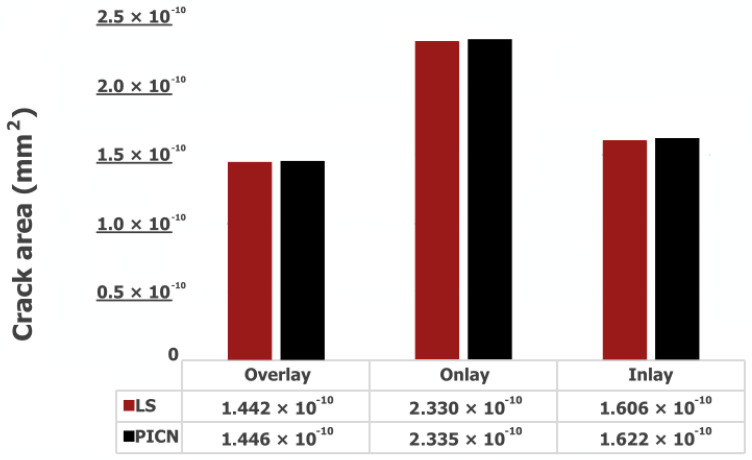
Area of cracks (mm^2^).

The propagation of the crack surface area with occlusal load for PPIR designs and materials is presented in Figure 4.

**Figure 5:**
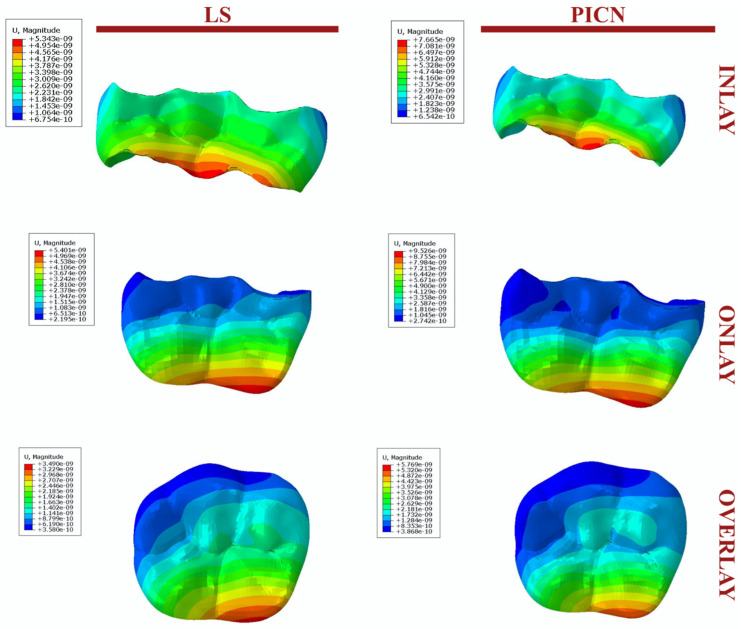
Displacement (U) values.

For different PPIR designs and materials, the restoration models with the most thickness demonstrated decreased crack surface area compared to the medium and least thickness models. This is also supported by the displacement (u) values. As the propagation of the crack initiates, conspicuous discontinuities manifest across all orientations of the crack front. Particularly noteworthy is the discernible movement exhibited by the lingual segment of the restoration. This dynamic behavior underscores the complex interplay of forces and stresses as the crack evolves (Figure 5).

**Figure 6:**
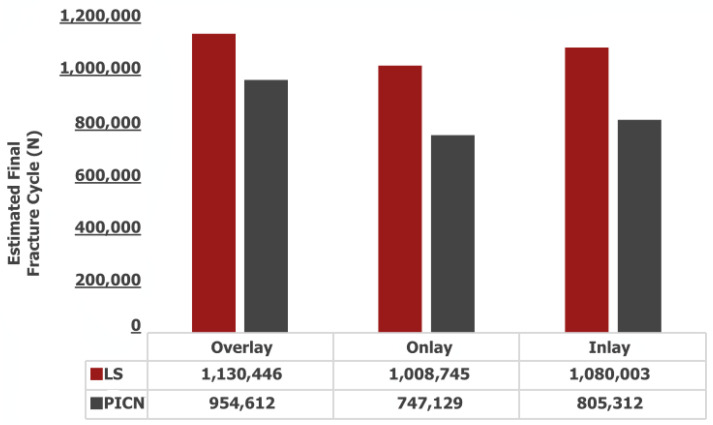
Estimated final fracture cyles.

Regarding the influence of material characteristics, the simulations revealed that PICNs exhibited a pronounced propensity for brittleness. Notably, following a span of 1000 fatigue cycles, a substantial variance in crack surface area became apparent when juxtaposed against the corresponding behavior of LS. This discernible disparity in crack propagation behavior underscores the distinct mechanical responses of these materials under cyclic loading conditions (Figure 4). Among the evaluated restoration designs, overlay restorations emerged as the most resilient in terms of fatigue performance, while, conversely, onlay restorations displayed comparatively inferior performance in this regard. Furthermore, it is noteworthy that across all variations of PPIR, the fatigue performance exhibited by LS materials surpassed that of the corresponding PICN materials. This consistent observation underscores the enhanced durability of LS materials within the context of each PPIR type (Figure 6).

## 4. Discussion

This study demonstrates that both the restoration type and restoration material affect the fatigue performance and progression patterns of cracks that form on restorations. Therefore, the initial hypothesis was rejected.

Finite element analysis is a dental biomechanical research method used to estimate stresses emerging in oral tissues and the clinical performance of restorative materials [33]. It is one of the best methods for simulating in vitro complex structures such as oral tissues. In this study, XFEM analysis was used to examine the crack propagation and fatigue performance of different CAD/CAM ceramic restorations and PPIRs. It was expected that the cracks occurred in three phases during fatigue performance: crack nucleation, crack propagation, and final fracture [28]. In this study, the crack nucleation phase was initiated by creating a standard initial crack on each PPIR type.

It was found that overlay restorations demonstrated the best fatigue performance, whereas onlay restorations were the least successful. Hofsteenge et al. reported that overlay ceramic restorations demonstrated better fracture resistance than inlay ceramic restorations with the same thickness [34]. However, Morimoto et al. suggested that there was no significant difference with regard to fracture resistance between inlay, onlay, and overlay restorations [35]. Using different materials, Wafaie et al. investigated the fracture resistance of inlay and onlay restorations and reported that inlay restorations fractured with bigger loads [36]. Cubas [37] and Habekost [38] support these findings. However, Huda et al. [39] and Kassis et al. [40] reported in their studies that onlay restorations demonstrated better fracture resistance compared to inlay restorations. Additionally, Yoon et al. investigated the fracture resistance of different MOD restoration types constructed based on different cavity preparation designs, and they were ranked from greatest to least resistance as overlay, onlay, and inlay. Therefore, there is no consensus in the literature as to the comparison of inlay, onlay, and overlay restorations in terms of fracture resistance [41]. Overlay restoration covers all cusps, and with this cusp coverage, the distribution of occlusal loads to the tooth surface and the concentration of stress in local areas can be prevented [42]. Therefore, the best fatigue performance might be attributable to overlay restorations. This also explains the fact that onlay restorations, in which there are few cusps covered, demonstrate less adequate fatigue performance. However, it is not sufficient to explain that inlay restorations demonstrate better fatigue performance than onlay restorations. It may simply be that inlay restorations encounter a smaller occlusal load compared to overlay and onlay restorations with similar antagonist contacts, and most of the occlusal load is transmitted directly to the dental tissues. In addition, minimum removal from the tooth structure may explain that inlay restorations demonstrate better fatigue performance than onlay types [36].

Upon evaluating the projected fracture cycles to assess durability, it becomes evident that overlay restorations exhibit the highest degree of resilience. Furthermore, when deliberating the merits of minimal tooth tissue removal during the contemplation of a Partial Posterior Indirect Restoration (PPIR) application, inlay restorations emerge as a sufficiently robust alternative. The superiority of overlay restorations stems from their notable ability to withstand fracture cycles, thereby affirming their durability. This durability is particularly noteworthy given the intricacies of PPIR, where preservation of tooth tissue is a paramount consideration. Inlay restorations, while involving a modest degree of tooth tissue removal, conversely display commendable endurance, making them a viable option in scenarios where conservative approaches are favored. Ultimately, these findings underscore the resilience of both overlay and inlay restoration designs, rendering them valuable choices in the spectrum of dental interventions (Figure 6).

**Figure 7:**
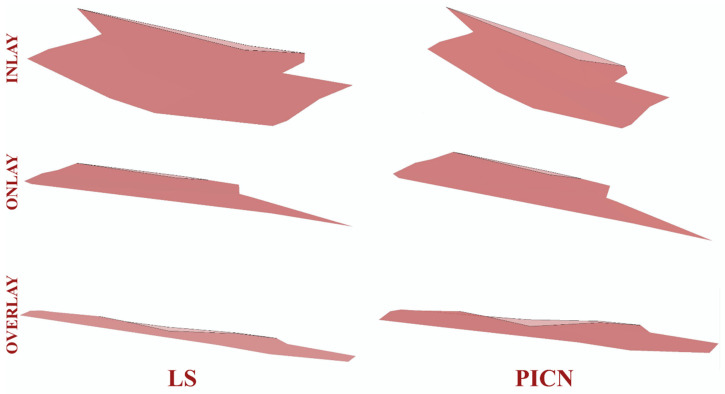
Propagation of cracks from a top view.

In this study, with each PPIR type, LS exhibited better fatigue performance than PICNs. In LS glass ceramics, crack propagation takes place in the glassy matrix. It is constantly deflected and creates an increase in volume within the matrix. This causes the interlocking of remaining crystal structures and causes the propagation of cracks to halt at the crack tip [43], as has been inarguably demonstrated in this XFEM study (Figure 7). In PICN materials, crack propagation occurs during the continuous reinforced glass phase, and the enmeshed resin matrix is responsible for bonding the progressive crack and re-establishing a linear path [44]. However, the crack in the resin matrix does not follow a linear path, so secondary cracking and plastic deformation may occur [45,46]. Although it is thought that the energy dissipated as a result of this phenomenon contributes to the secondary toughness of the resin-containing materials, this effect may not be as strong as in the crystal structures of LS glass ceramics.

In this study, the greatest displacement was observed in onlay restorations and the least in overlay restorations. These results are consistent with the fatigue performance described already. Moreover, in terms of the fatigue test, the regions where the cracks spread demonstrated a similar distribution. The total cracked area was highest in the onlay restorations and lowest in the overlay restorations (Figure 4).

In this study, the crack propagation pattern observed in the LS material did not only follow a linear path. After the crack continued to form, it expanded in all directions and re-established a linear path. A situation similar to the crack propagation pattern in the LS material occurred in the PICN material: as the crack continued to grow, it expanded in all directions and re-established a linear path. However, the displacement of the crack in all directions before it refollowed a linear path covered a wider area than in the LS material (Figure 7). Unlike ductile materials, brittle materials exhibit a more direct crack propagation pattern [28]. As observed in this study, in the LS material, the cracks were less displaced in the crystal structure, while in PICN materials, the cracks were displaced more during the continuous reinforced glass phase.

In this study, the periodontal ligament (PDL) was not simulated. This approach is consistent with several previous studies [47,48]. The simulation of the PDL in finite element modeling (FEM) remains a debated topic due to the difficulties in standardizing its thickness [49] and the challenges in simulating its time- and direction-sensitive response to strain results induced by load [50]. Additionally, Abraha et al. reported in their study that both simulations with and without the PDL yielded results consistent with values from their in vivo studies [50].

The limitation of this study is that the material properties could not have been prepared realistically through finite element analysis. It was supposed that the restorations and teeth bound to each other perfectly and all possible errors in material production were ignored. Moreover, 1000 cycles were used for fatigue performance. Increasing the number of cycles could have yielded more comprehensive results. In the future, it is recommended that validation studies supported by real cyclic studies be performed and a higher number of cycles applied.

## 5. Conclusions

In scenarios necessitating the application of partial posterior indirect restoration (PPIR), it is conceivable that LS glass ceramics may manifest superior performance relative to PICN materials. Furthermore, within the domain of PPIRs, approaches centered around inlays are poised to achieve a higher likelihood of favorable outcomes, particularly when the phenomenon of maximal tissue preservation is taken into account. Given the extant clinical conditions, in cases mandating cuspal coverage, the implementation of an overlay restoration emerges as a more judicious choice, contrasting with onlay restorations.

## Figures and Tables

**Table 1:** Mechanical properties of materials.

Materials	Young’s Modulus (MPa)	Poisson’s Ratio	Compressive Strength (Mpa)	Tensile Stress σ_TS_ (Mpa)	Fracture Toughness K_IC_ (Mpa × m12)	Strain Energy Release Rate G (J/m^2^)	References
Dentin	18,600	0.31	282	-	-	-	[1]
Enamel	84,000	0.33	321	-	-	-	[1]
LS	98,000	0.23	-	210	2.27	52.6	[2]
PICN	35,000	0.23	-	148	1.2	41.1	[2]
RelyX ARC	5100	0.27	-	-	-	-	[3]

## Data Availability

The data presented in this study are available on request from the corresponding author.
